# Sex Differences in Neural Correlates of Emotion Regulation in Relation to Resting Heart Rate Variability

**DOI:** 10.1007/s10548-023-00974-9

**Published:** 2023-06-23

**Authors:** Jungwon Min, Julian Koenig, Kaoru Nashiro, Hyun Joo Yoo, Christine Cho, Julian F. Thayer, Mara Mather

**Affiliations:** 1 University of Southern California, Davis School of Gerontology, Los Angeles, USA; 2 University of Southern California, Department of Psychology, Los Angeles, USA; 3 University of Cologne, Faculty of Medicine and University Hospital Cologne, Department of Child and Adolescent Psychiatry, Psychosomatics and Psychotherapy, Cologne, Germany; 4 University of California Irvine, Department of Psychological Science, Irvine, USA

**Keywords:** Emotion regulation, fMRI, Heart rate variability, Neuroimaging, RMSSD, Sex difference

## Abstract

Prior studies suggest that sex differences in emotion regulation (ER) ability contribute to sex disparities in affective disorders. In behavioral studies, females rely more on maladaptive strategies to cope with emotional distress than males. Neuroimaging studies suggest that males more efficiently regulate emotion than females by showing less prefrontal cortex activity (suggesting less effort) for similar amygdala activity (similar regulation outcome). However, physiological studies involving heart rate variability (HRV) indicated that, compared with males, females have higher resting HRV, indicative of parasympathetic dominance and better control of emotion. To help resolve these apparently inconsistent findings, we examined sex differences in how resting HRV relates to brain activity while using cognitive reappraisal, one of the adaptive strategies. Based on 51 males and 49 females, we found that females showed different levels of self-rated emotional intensity and amygdala activity for negative versus positive emotions, while males did not. Females also showed greater overall prefrontal cortex activity but similar levels of amygdala activity compared to males. Sex differences in how resting HRV related to brain activity during ER were evident only during viewing or regulating positive emotion. The results suggest that sex differences in the neural correlates of ER and resting HRV might lie in valence more than arousal modulation.

## Introduction

Men and women differ in many aspects of emotion processing (Kret and de Gelder 2012; Vigil 2009; Whittle et al. 2011). Compared to men, women are better at decoding affective cues (Hall et al. 2016; Hampson et al. 2006), are more reactive to emotional stimuli (Lithari et al. 2010; Proverbio et al. 2009), and more frequently express sadness and fear (Safdar et al. 2009). In contrast, men relative to women are more sensitive to dominance-related stimuli (Vigil 2008) and more likely to display aggressive behavior (Archer 2004). These differences do not always consistently predict psychological outcomes. Although females outperform males in emotional intelligence tests (Joseph and Newman 2010), they are more susceptible to affective disorders such as generalized anxiety and major depression (Altemus et al. 2014).

Prior studies point to sex differences in emotion regulation (ER) ability as a key factor to explain these inconsistencies (Kelly et al. 2008; Thayer et al. 2003). One prominent difference is that females, compared to males, tend to gravitate towards maladaptive strategies (e.g., rumination, avoidance, and wishful thinking) to cope with emotional distress (Johnson and Whisman 2013; Matud 2004; Tamres et al. 2002). Although females more flexibly choose from multiple ER strategies involving both adaptive and maladaptive strategies (Goubet and Chrysikou 2019; Tamres et al. 2002), the negative impact of maladaptive strategies seems to exceed the beneficial effect of adaptive strategies, leading to psychiatric conditions. Use of maladaptive strategies (e.g., avoidance and suppression) was associated with the occurrence of anxiety and depression with a large effect size, while use of adaptive strategies (e.g., acceptance and reappraisal) was negatively associated with their occurrence with a small effect size (Aldao et al. 2010).

Neuroimaging studies suggest sex differences in neural systems involved in ER might contribute to the disparity in the affective disorders. Yet, findings of functional magnetic resonance imaging (fMRI) studies using blood oxygenation level dependent (BOLD) signals do not provide a straightforward understanding (Whittle et al. 2011). In one study, during regulating negative emotion, females relied on the medial orbitofrontal cortex, while males recruited the dorsolateral prefrontal cortex (Mak et al. 2009). However, in another study, males recruited the prefrontal cortex (PFC) less, but decreased amygdala activity more than females while decreasing negative emotion compared to viewing (McRae et al. 2008). Based on this, McRae et al. proposed a hypothesis that ER in males might be more efficient because they exert less cognitive effort represented by PFC activity while more effectively reducing arousal levels represented by amygdala activity. However, this efficiency framework does not seem consistent with other findings where males showed stronger PFC activity than females with no sex difference in amygdala activity during emotion down-regulation (Domes et al. 2010). Besides small sample sizes (24–33 participants), these mixed findings might be due to the studies’ different task designs that covered partial dimensions of valence and arousal. While Domes et al.’s study focused on the down- and up-regulation of negative emotion (2010), the other two studies were based on the down-regulation of negative and positive emotions (Mak et al. 2009; McRae et al. 2008). To obtain more generalizable findings, it is necessary to design a study accommodating both up- and down-regulation of negative and positive emotions with a larger sample size.

Another line of research has examined how ER is related to different patterns of physiological activity. Heart rate variability (HRV) is a physiological measure that reflects ER ability; higher HRV at rest is associated with better ER ability (Thayer and Lane 2009). For healthy adults, HRV is associated with positive mood, and the association was mediated by habitual use of cognitive ER strategies (Geisler et al. 2010). Higher HRV also works as a buffer by reducing the negative consequences of ER difficulties (Fantini-Hauwel et al. 2020). On the other hand, people with depression and anxiety have lower HRV than those without the conditions (Chalmers et al. 2014; Koch et al. 2019). As females are more likely to experience anxiety and depression than males (Altemus et al. 2014), a reasonable prediction would be that females have lower HRV than males. However, converging evidence suggests the opposite. A meta-analysis involving 172 studies indicated that females showed higher resting HRV than males (e.g., greater power in high-frequency band), indicative of greater parasympathetic activity and better control of emotion (Koenig and Thayer 2016).

Even though multiple studies investigated ER in relation to either sex or HRV, we found no neuroimaging studies that examined sex differences in how brain activity during ER relates to resting HRV. Although one fMRI study demonstrated that individuals with higher HRV more flexibly recruit medial PFC (mPFC) and better modulate the amygdala activity during cognitive reappraisal, it did not address sex differences (Steinfurth et al. 2018). To the extent of our knowledge, the current study is the first fMRI study that investigates sex differences in the relationship between resting HRV and brain activity during ER. We predict that PFC and amygdala activity during ER would differ between males and females but without a specific direction because of the inconsistent findings (Domes et al. 2010; McRae et al. 2008). Males could more flexibly recruit the PFC to modulate amygdala activity based on females’ tendency to use maladaptive ER strategies (Johnson and Whisman 2013; Matud 2004; Tamres et al. 2002), but the same prediction is equally possible for females as they show parasympathetic dominance relative to males (Koenig and Thayer 2016). Focusing on the PFC and amygdala could miss other sex-specific brain regions associated with resting HRV and ER. Thus, in addition to the amygdala and PFC region of interest (ROI) analyses, we ran a whole brain analysis.

## Materials and Methods

### Participants

The current study is based on the ER task completed during the pre-intervention phase of a HRV biofeedback intervention study (ClinicalTrials.gov Identifier: NCT03458910), approved by University of Southern California’s Institutional Review Board. In the intervention, participants were randomly assigned to daily practice sessions where they practiced different breathing techniques either to increase or decrease their heart rate oscillations (Nashiro et al. 2022). We recruited participants without serious medical or psychiatric illness via USC’s subject pool, USC’s online bulletin board, Facebook, and flyers. Participants signed informed consent forms before beginning and were paid $15 per hour for their lab visits upon completion. The basic findings on the pre-intervention ER data were already published (Min et al. 2022), and the current study was initially based on the same number of participants (N = 105). Among the participants, we excluded three participants who did not have HRV measures at pre-intervention and two participants who had too extreme root mean square of successive differences (RMSSD) values (RMSSD> 200 ms) when considering their relatively small mean time-intervals between heart beats. Thus, we analyzed the data of 100 participants, consisting of 51 males and 49 females and aged from 18 to 31 years (M_age_ = 22.81, SD_age_ = 2.75).

### Task and Procedure

The task was based on a previously published ER task where participants were instructed to use cognitive reappraisal strategy to down- or up-regulate emotion for positive and negative emotions (Kim and Hamann 2007). The task lasted for 10 min with 42 trials. Each trial consisted of three phases: instruction, regulation, and rating in order (Min et al. 2022). During the 1-second instruction, participants were presented with “intensify”, “diminish”, or “view”. During the 6-second regulation, they down-regulated, up-regulated, or passively experienced emotions elicited by the presented images, which were negative, positive, or neutral. During the 4-second rating following each regulation phase, they rated the strength of their feeling in the moment (levels of emotional intensity) with an ascending order of 1 to 4.

These event trials were organized in a block-wise manner where three events of the same condition were contained in each block and separated by a fixation cross lasting for a varying interval. The two intervals separating three events within each block added up to 4 s. The blocks were separated by a 5-second fixation cross display and presented in a pseudorandom order such that participants did not experience two blocks with the same instruction and image valence in a row. We selected six sets of images with the same average valence (*M*_negative_ = 2.3, *M*_positive_ = 7.2, *M*_neutral_ = 5.0) and same arousal scores (*M*_negative_ = 5.4, *M*_positive_ = 5.4, *M*_neutral_ = 2.8) from the International Affective Picture System (Lang et al. 2008). Each set consisted of 18 negative, 18 positive, and 6 neutral pictures, which were presented in a pseudo-randomized order. There were seven regulation task conditions: diminish-negative, view-negative, intensify-negative, diminish-positive, view-positive, intensify-positive, and view-neutral.

We first administered emotion-related questionnaires assessing anxiety and depression levels (Nashiro, et al. 2022). State Trait Anxiety Inventory (STAI) and Center for Epidemiological Studies-Depression (CES-D) were collected on the same day before the MRI scans (Barnes et al. 2002; Radloff 1977). Before their MRI scans, participants were encouraged to employ their own reappraisal strategies and practiced for all seven types of trials. They were given examples of cognitive reappraisal strategies such as reinterpreting the situation or modifying their distance from the scene. They were also advised to avoid generating an opposite emotion to diminish the negative or positive emotion. After the scan, we asked participants to describe their regulation strategies and how successful they felt for each regulation task condition.

### MRI Data Acquisition

We collected MRI data at USC’s Dana and David Dornsife Cognitive Neuroimaging Center using a 3T Siemens MAGNETOM Prisma MRI scanner with a 32-channel head coil (Min et al. 2022). We obtained a T1-weighted MPRAGE anatomical image (TR = 2300 ms, TE = 2.26 ms, slice thickness = 1.0 mm, flip angle = 9°, field of view = 256 mm, voxel size = 1.0 isotropic). We acquired 250 whole brain volumes of T2*-weighted functional images using multi-echo planar imaging sequence (TR = 2400 mm, TE 18/35/53 ms, slice thickness = 3.0 mm, flip angle = 75°, field of view = 240 mm, voxel size = 3.0 isotropic).

### HRV Data Acquisition and Processing

On the same day as the MRI scan, heartbeat data were obtained from participants sitting in a chair with spontaneous breathing at the research lab for five minutes during daytime (Nashiro et al. 2022). Using HeartMath emWave Pro software and its infrared pulse plethysmograph (PPG) ear sensor, the heartbeat data was sampled at 370 Hz and its inter-beat interval data was recorded after emWave’s artifact removal. The inter-beat interval data was entered into Kubios HRV Premium 3.1 software to compute mean heart rate and RMSSD for each participant.

### Behavioral and HRV Data Analysis

We averaged the emotional intensity ratings separately for the seven regulation task conditions. To examine sex differences in self-reported emotional intensity ratings, we used SPSS (IBM, version 28) to test a mixed analysis of variance (ANOVA) model having 2 (valence: negative, positive) × 3 (regulation: diminish, view, intensify) × 2 (sex: male, female) factors. This ANOVA model included all regulation task conditions except the baseline view-neutral condition. We also used a similar ANOVA model to test sex differences in emotional intensity during passively viewing emotional images while controlling for emotional intensity during viewing neutral images. We ran independent sample t-tests to compare HRV measures including mean heart rate and RMSSD between males and females. We used the same t-tests to ensure that males and females do not differ in anxiety (STAI) and depression (CES-D) levels.

### MRI Data Analysis

We performed multi-echo independent component analysis to remove artifact components from time series data based on the linear echo-time dependence of BOLD signal fluctuations (Kundu et al. 2012). After denoising the data, we used FMRIB Software Library (FSL) version 6.0 for the individual- and group-level analysis (Jenkinson et al. 2012; Woolrich et al. 2001, 2004). During the individual-level analysis, each functional image was registered to the MNI152 T1 2 mm template via its T1-weighted anatomical image using affine linear transformation with 12 degrees of freedom. Individual-level analysis also included a preprocessing of motion correction, spatial smoothing with 5 mm FWHM, and high-pass filtering with 600-second cutoff.

To examine which brain regions’ activity during ER correlated with individual differences in resting HRV, we modeled individual whole-brain BOLD time-series by setting the seven emotion-regulation regressors during the 6-second regulation phase (diminish-negative, view-negative, intensify-negative, diminish-positive, view-positive, intensify-positive, and view-neutral) along with their temporal derivatives, each of which were convolved with a double-gamma hemodynamic response function. To examine how males and females differ in brain activity during ER as well as in the relationship between resting HRV and the brain activity, the group-level analysis was set to have four regressors, two for males and females and two for males and females’ demeaned RMSSD using FSL’s mixed-effects model (FLAME 1). The final results were corrected for family-wise error at *p* < 0.05 with the cluster-size threshold at *z* > 2.3.

In addition to the whole-brain analysis, we performed ROI analysis by focusing on two brain regions, the amygdala and PFC, whose activities are involved in HRV as well as ER (Thayer and Lane 2009). To assess BOLD activity changes in the amygdala, we individually segmented the amygdala region from each participant’s T1-weighted image using FreeSurfer version 6 (Fischl et al. 2002, 2004) and created the left and right amygdala masks in the native space. We then applied FSL FLIRT (Jenkinson et al. 2002; Jenkinson and Smith 2001) to transform the masks to the standard MNI space and input them to Featquery. Using *Featquery*, we extracted each participant’s percent signal change values in bilateral amygdala activity for each condition (diminish-negative, view-negative, intensify-negative, diminish-positive, view-positive, intensify-positive, and view-neutral). To assess BOLD activity in the PFC region, we created a binary mask by taking the prefrontal area of the clusters which were activated during both the diminish and intensify conditions in our prior findings (Min et al. 2022; Fig. 5A, mostly the left dorsolateral PFC). Similar to the amygdala, we used *Featquery* to obtain average percent signal changes in the PFC mask region for each condition.

As prior fMRI studies compared BOLD activity in the amygdala and PFC to address sex differences in the effect of ER (Domes et al. 2010; McRae et al. 2008), we compared changes in the two regions during ER. We first used ANOVA to test sex differences in BOLD signal changes in the bilateral amygdala as well as PFC during viewing negative or positive images while controlling for viewing neutral images. We then examined whether each region's activity differed between males and females based on the valence and regulation conditions by running mixed 2 × 3 × 2 ANOVA, (negative, positive) × (diminish, view, intensify) × (male, female).

## Results

### Self-Rated Emotional Intensity and HRV

We first tested sex differences in the self-rated emotional intensity during viewing emotional images while controlling for emotional intensity during viewing neutral images. There was a significant main effect of valence, *F*(1, 97) = 8.27, *p* = 0.01, Δ*η*^2^ = 0.08 (M = 2.32 and SE = 0.06 for viewing negative emotion; M = 2.18 and SE = 0.06 for viewing positive emotion). There was no main effect of sex, *F*(1, 97) = 0.75, *p* = 0.39, Δ*η*^2^ = 0.01 (M = 2.20 and SE = 0.07 for males; M = 2.29 and SE = 0.07 for females). There was no interaction effect between sex and valence on the emotional intensity during viewing emotional images, *F*(1, 97) = 0.91, *p* = 0.34, Δ*η*^2^ = 0.01.

**Fig. 1:**
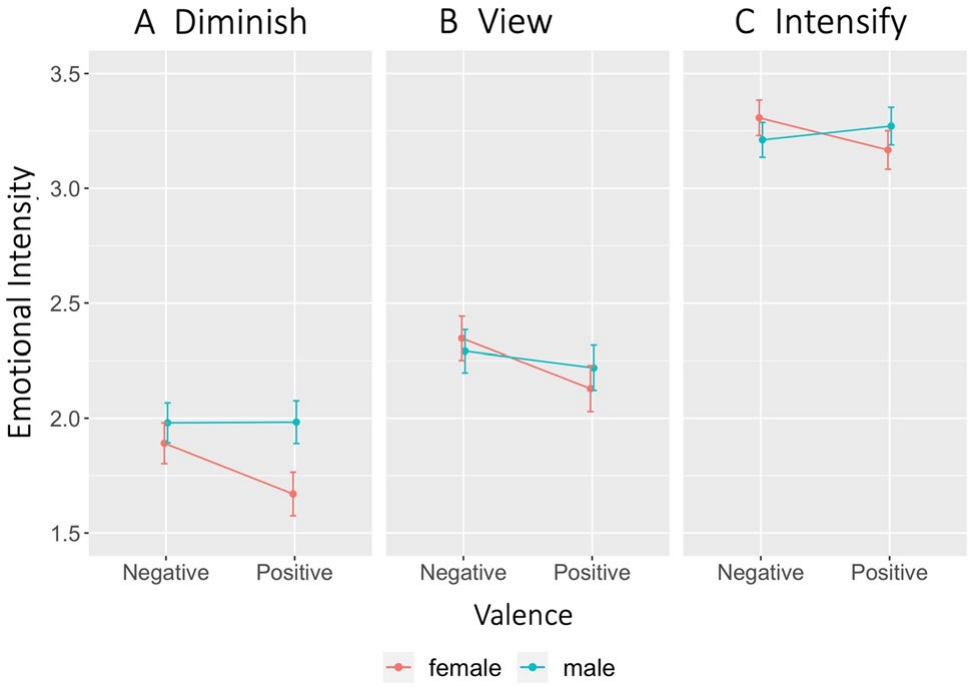
Sex difference in emotional intensity ratings for valence and regulation. Females differently rated emotional intensity for negative and positive emotions, while males did not show a difference. Males and females showed similar patterns across the three regulation conditions. Error bars represent standard errors

We then tested sex differences in the emotional intensity ratings during ER using a mixed 2 (valence: negative, positive) × 3 (regulation: diminish, view, intensify) × 2 (sex: males, females) ANOVA model. There was no significant main effect of sex, *F*(1, 98) = 0.78, *p* = 0.38, Δ*η*^2^ = 0.01 (M = 2.49 and SE = 0.06 for males; M = 2.42 and SE = 0.06 for females). In addition, the main effects of valence and regulation were significant, *F*(1, 98) = 5.64, *p* = 0.02, Δ*η*^2^ = 0.05 for valence (M = 2.51 and SE = 0.04 for negative emotion; M = 2.41 and SE = 0.05 for positive emotion) and *F*(1, 98) = 215.33, *p* < 0.001, Δ*η*^2^ = 0.69 for regulation (M = 1.88 and SE = 0.06 for diminish; M = 2.25 and SE = 0.06 for view; M = 3.24 and SE = 0.05 for intensify). In the same model, there was no significant 2 × 3 interaction effect between sex and regulation, *F*(2, 196) = 1.32, *p* = 0.27, Δ*η*^2^ = 0.01, but we found a significant 2 × 2 interaction effect between sex and valence, *F*(1, 98) = 5.28, *p* = 0.02, Δ*η*^2^ = 0.05. We found that males and females differently reported emotional intensity for negative and positive emotions (Fig. 1A–C). As indicated by the means and the 95% CIs, females reported greater emotional intensity in negative emotion (M = 2.52, 95% CI [2.39, 2.64]) than positive emotion (M = 2.32, 95% CI [2.18, 2.46]), while males rated negative (M = 2.49, 95% CI [2.37, 2.61]) and positive emotions (M = 2.49, 95% CI [2.35, 2.63]) similarly (Fig. 1). There was no 3-way interaction effect of valence × regulation × sex, *F*(2, 196) = 0.21, *p* = 0.81, Δ*η*^2^ = 0.002. We did not find a simple effect of sex for either valence, *p* = 0.27 for negative emotion and *p* = 0.06 for positive emotion. Mean ratings and 95% confidence intervals for males and females in all seven conditions are reported in Table 1. We did not find significant sex differences in RMSSD, *t*(98) = − 0.46, *p* = 0.64 and mean heart rate, *t*(98) = − 1.05, *p* = 0.30, while we did see sex differences in high and low-frequency power (Table 2). There were no sex differences in state anxiety, *t*(98) = -1.37, *p* = 0.17, trait anxiety, *t*(98) = -1.18, *p* = 0.24, and depression levels, *t*(98) = -1.77, *p* = 0.08.

**Table 1:** Sex differences in emotional intensity ratings during emotion regulation

	Males (N = 51)			Females (N = 49)		
	M	SE	95% CI	M	SE	95% CI
Diminish negative	1.98	0.09	[1.79, 2.16]	1.89	0.08	[1.72, 2.06]
View negative	2.29	0.09	[2.10, 2.48]	2.35	0.10	[2.15, 2.54]
Intensify negative	3.21	0.09	[3.04, 3.38]	3.31	0.07	[3.17, 3.44]
Diminish positive	1.98	0.10	[1.77, 2.19]	1.67	0.08	[1.50, 1.84]
View positive	2.22	0.11	[2.00, 2.44]	2.13	0.09	[1.95, 2.30]
Intensify positive	3.27	0.09	[3.09, 3.45]	3.17	0.07	[3.02, 3.32]
View neutral	1.52	0.10	[1.33, 1.71]	1.33	0.08	[1.17, 1.49]

**Table 2:** Sex differences in resting HRV measures

	Males		Females		Statistics			
	M	SE	M	SE	*t*	*df*	*2-tailed p*	95% CI of difference
RMSSD (ms)	59.70	4.32	62.47	4.14	− 0.46	98	0.64	[− 14.65, 9.11]
Mean heart rate (bpm)	71.67	1.25	73.69	1.48	− 1.05	98	0.30	[− 5.86, 1.81]
Mean heart period (ms)	850.47	15.35	830.95	17.73	0.83	98	0.41	[− 26.91, 65.94]
Low frequency power (nu)	58.23	2.26	50.64	2.46	2.27	98	0.03	[0.96, 14.21]
High frequency power (nu)	41.66	2.25	49.18	2.46	− 2.26	98	0.03	[− 14.13, − 0.92]

### Sex Differences in Amygdala and PFC BOLD Activity During Emotion Regulation

We compared amygdala activity between males and females during viewing negative and positive images when controlled for viewing neutral images. While we did not find a significant main effect of valence or sex in bilateral amygdala BOLD activity (all *p*s > 0.16), we found a significant interaction between sex and valence, *F*(1, 97) = 5.36, p = 0.02, Δ*η*^2^ = 0.05. While females did not show any difference between viewing negative and positive (*p* = 0.56), males showed greater amygdala activity during viewing negative images than positive images (*p* = 0.01). Amygdala activity for males and females in all seven conditions are reported in Table 3.

**Table 3:** Sex differences in amygdala and PFC activity during emotion regulation

	Males (N = 51)			Females (N = 49)		
	M	SE	95% CI	M	SE	95% CI
Amygdala						
Diminish negative	− 0.004	0.009	[− 0.022, 0.014]	0.011	0.010	[− 0.009, 0.031]
View negative	0.036	0.007	[0.022, 0.050]	0.011	0.014	[− 0.017, 0.040]
Intensify negative	0.038	0.013	[0011, 0.065]	0.060	0.012	[0.036, 0.085]
Diminish positive	0.023	0.010	[0.002, 0.044]	− 0.006	0.011	[− 0.028, 0.015]
View positive	− 0.000	0.011	[− 0.023, 0.023]	0.018	0.010	[− 0.002, 0.038]
Intensify positive	0.052	0.010	[− 0.032, 0.073]	0.026	0.012	[0.003, 0.050]
View neutral	0.002	0.010	[− 0.018, 0.022]	− 0.011	0.011	[− 0.034, 0.011]
Prefrontal cortex						
Diminish negative	0.015	0.015	[− 0.015, 0.046]	0.067	0.016	[0.035, 0.098]
View negative	0.010	0.013	[− 0.016, 0.038]	0.028	0.014	[− 0.000, 0.056]
Intensify negative	0.043	0.013	[0.016, 0.070]	0.087	0.014	[0.058, 0.116]
Diminish positive	0.048	0.014	[0.027, 0.075]	0.074	0.013	[0.047, 0.101]
View positive	− 0.015	0.015	[− 0.046, 0.016]	0.057	0.012	[0.033, 0.081]
Intensify positive	0.055	0.013	[0.029, 0.082]	0.091	0.016	[0.059, 0.122]
View neutral	− 0.016	0.011	[− 0.038, 0.005]	0.007	0.014	[− 0.022, 0.035]

**Fig. 2:**
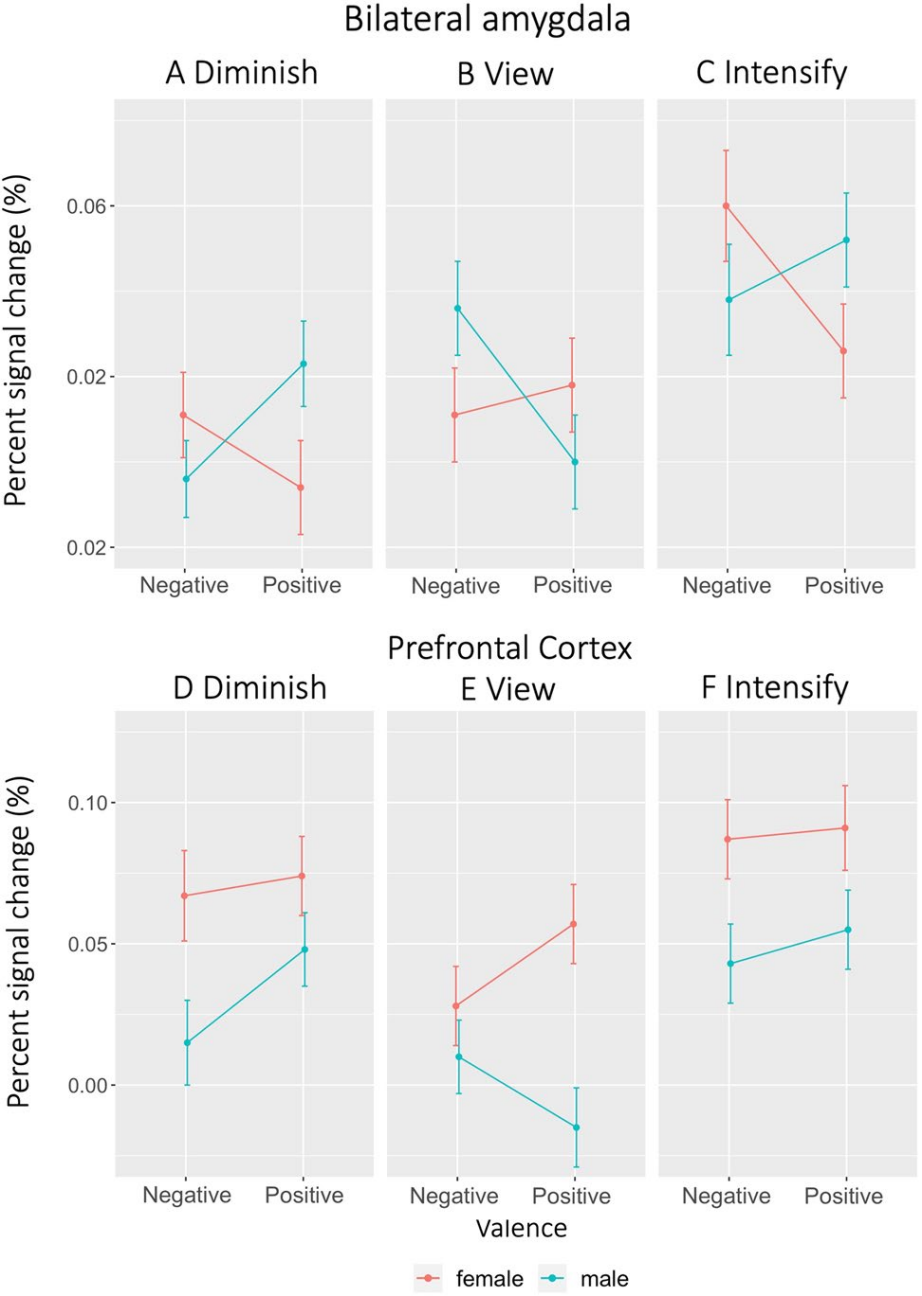
Amygdala and PFC BOLD activity of males and females during emotion regulation. Females and males showed different patterns across the negative and positive valence for each regulation condition (A–C). Females showed overall higher PFC activity relative to males for both the valence and regulation conditions (D–E). Error bars represent standard errors

We next ran a mixed ANOVA to test sex differences in amygdala BOLD activity based on valence (negative, positive) and regulation (diminish, view, intensify). While the main effect of valence or sex was not significant (all *p*s > 0.1), the main effect of regulation was significant, *F*(2, 196) = 18.77, *p* < 0.001, Δ*η*^2^ = 0.16. In the same model, we found that there was no significant interactions between regulation and sex, *F*(1.83, 178.97) < 0.001, *p* = 0.90, Δ*η*^2^ = 0.001, but there was a significant interaction between valence and sex, *F*(1, 98) = 4.21, *p* = 0.04, Δ*η*^2^ = 0.04. Males showed similar amygdala activity for negative (M = 0.02, SE = 0.01, 95% CI [0.01, 0.04]) and positive (M = 03, SE = 0.01, 95% CI [0.01, 0.04]) emotions, while females showed greater amygdala activity in negative (M = 0.03, SE = 0.01, 95% CI [0.01, 0.04]) than positive (M = 0.01, SE = 0.01, 95% CI [− 0.002, 0.03]) emotion. We also found a 3-way interaction of valence x regulation x sex in amygdala activity, *F*(1.74, 170.07) = 6.60, *p* = 0.003, Δ*η*^2^ = 0.06 (Fig. 2A–C).

Like for the amygdala, we also tested sex differences in PFC BOLD activity during viewing negative and positive images when controlled for PFC activity during viewing neutral images. There was no main effect of valence, *F*(1, 97) = 0.07, p = 0.79, Δ*η*^2^ = 0.001, but there was a main effect of sex in PFC activity, *F*(1, 97) = 6.33, p = 0.01, Δ*η*^2^ = 0.06, M = 0.003, 95% CI [− 0.02, 0.02] for males and M = 0.04, 95% CI [0.02, 0.06] for females. We also found a valence x sex interaction, *F*(1, 97) = 4.93, p = 0.03, Δ*η*^2^ = 0.05. While males and females did not differ in their PFC activity for viewing negative images (*p* = 0.65), females showed greater PFC activity levels than males for viewing positive images (*p* < 0.001).

We next ran a mixed ANOVA to test sex differences in PFC BOLD activity based on valence (negative, positive) and regulation (diminish, view, intensify). There were significant main effects of valence, *F*(1, 98) = 4.14, *p* = 0.05, Δ*η*^2^ = 0.04, and regulation, *F*(2, 196) = 23.04, *p* < 0.001, Δ*η*^2^ = 0.19. There was also significant main effect of sex, *F*(1, 98) = 7.58, *p* = 0.01, Δ*η*^*2*^ = 0.07, indicating overall greater PFC activity for females than males (Fig. 2D–F; Table 3). We found no interaction in PFC activity between regulation and sex, *F*(2, 196) = 0.09, *p* = 0.91, Δ*η*^2^ = 0.001 as well as between valence and sex, *F*(1, 98) = 0.46, *p* = 0.50, Δ*η*^2^ = 0.01. However, there was a 2 × 3 × 2 interaction, *F*(1.80, 176.69) = 3.69, *p* = 0.03, Δ*η*^2^ = 0.04.

To investigate whether males and females showed different ER outcomes for negative and positive emotions in terms of changes in amygdala BOLD activity, we ran post-hoc analyses with Bonferroni correction (corrected alpha level = 0.006). Males and females showed different patterns in amygdala activity change for negative and positive emotions. During diminishing negative emotion compared with viewing, males decreased amygdala activity, M_Difference_= − 0.04, 95% CI_Difference_ = [− 0.064, − 0.016], *p* = 0.001, but females did not, M_Difference_ = − 0.001, 95% CI_Difference_ = [− 0.025, 0.024], *p* = 0.96. During intensifying negative emotion compared to viewing, females increased amygdala activity, M_Difference_ = 0.05, 95% CI_Difference_ = [0.018, − 0.079], *p* = 0.002 (Fig. 2A–C), but males did not, 0.002, 95% CI_Difference_ = [− 0.028, 0.032], *p* = 0.089.

When it comes to diminishing positive emotion compared to viewing, males and females did not decrease amygdala activity, M_Difference_ = 0.02, 95% CI_Difference_ = [− 0.006, 0.051], *p* = 0.11 for males and M_Difference_ = − 0.02, 95% CI_Difference_ = [− 0.053, 0.005], *p* = 0.10 for females. During intensifying positive emotion compared to viewing, males increased amygdala activity, M_Difference_ = 0.052, 95% CI_Difference_ = [0.027, 0.078], *p* < 0.001, but females did not show a significant change, M_Difference_ = 0.008, 95% CI_Difference_ = [− 0.018, 0.035], *p* = 0.53. Even though many of the pairs did not show significant differences between conditions for positive emotion, we note that females showed changes in amygdala activity according to the regulation condition such that the activity level was lowest for diminishing and highest for intensifying. However, amygdala activity in males was not consistent with regulation conditions because it was lowest for viewing and increased for diminishing positive emotion (Fig. 2A–C).

### Sex Differences in Whole-Brain BOLD Activity During Emotion Regulation

**Fig. 3:**
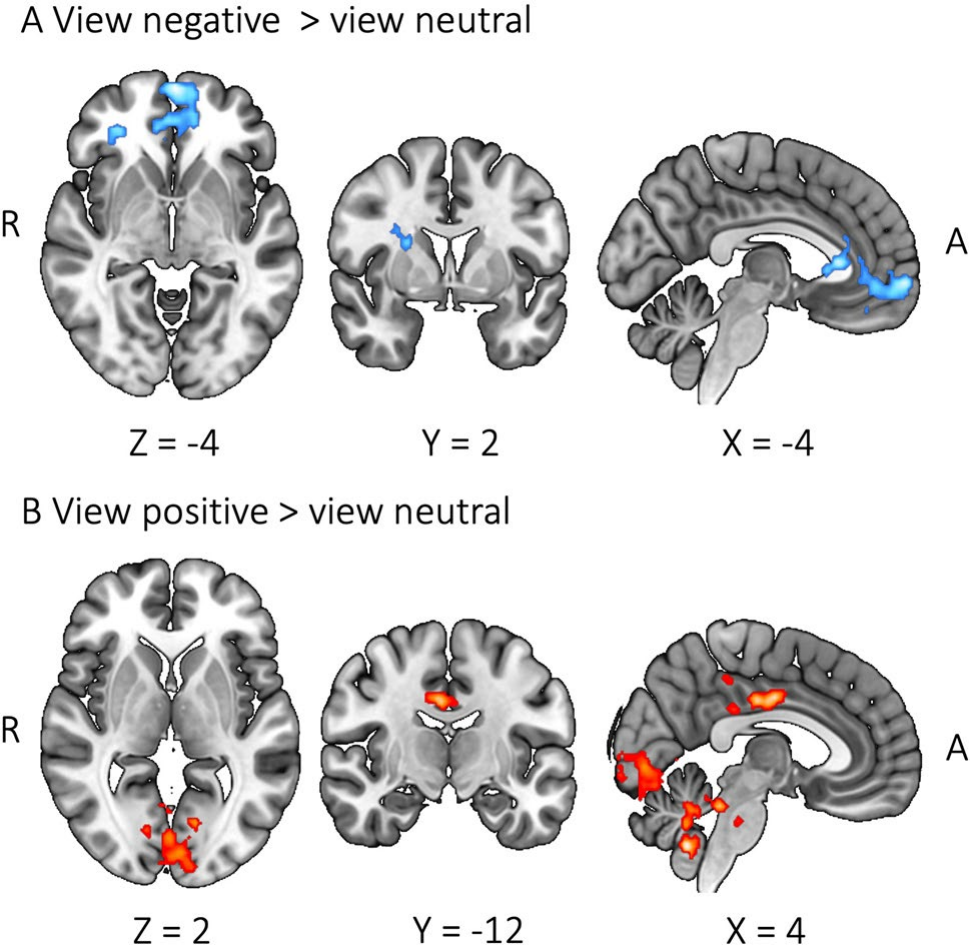
Sex differences during viewing emotional images. Blue color displays males > females; red color displays females > males

We first examined which brain regions differed between males and females when just viewing emotional images. Contrasting viewing negative images against viewing neutral images revealed that males increased BOLD activity relative to females in the frontal pole and frontal medial cortex (Fig. 3A). For the same contrast, females compared to males did not show any significant clusters. Viewing positive images against neutral images showed that females increased BOLD activity compared to males in the posterior cingulate gyrus (PCC), lingual gyrus, occipital pole, and cerebellum (crus II and vermis IX) (Fig. 3B). No significant clusters were found for males versus females during viewing positive images against neutral ones.

**Fig. 4:**
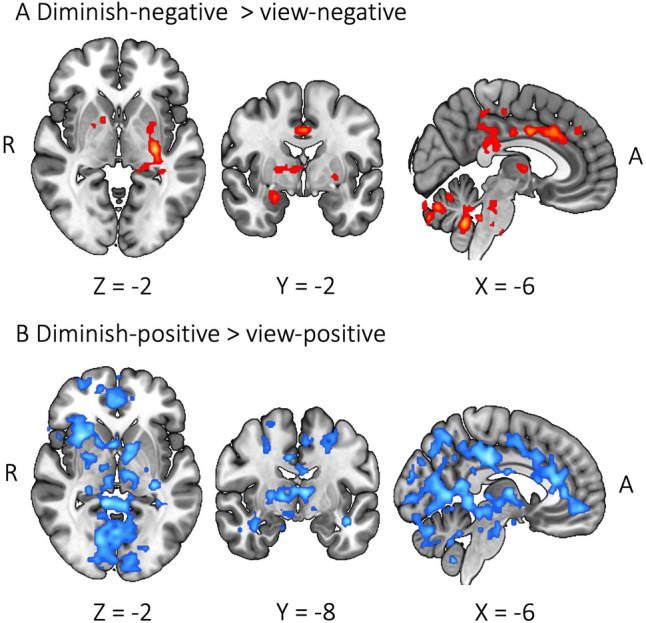
Sex differences during emotion down-regulation. Blue color displays males > females; red color displays females > males

Males and females also showed differences when down-regulating emotion compared to viewing. The contrast of diminishing negative emotion against viewing revealed that females versus males showed increased BOLD activity in the anterior cingulate gyrus (ACC), PCC, thalamus, putamen, caudate, and cerebellum (VI, crus I, crus II) (Fig. 4A). The same contrast for males versus females did not show significant clusters. When diminishing positive emotion against viewing, males compared to females showed increased BOLD activity in broad regions including insula, paracingulate gyrus, ACC, PCC, lingual gyrus, brainstem, thalamus, and pallidum (Fig. 4B). No significant clusters were found for females compared to males in the same contrast.

**Fig. 5:**
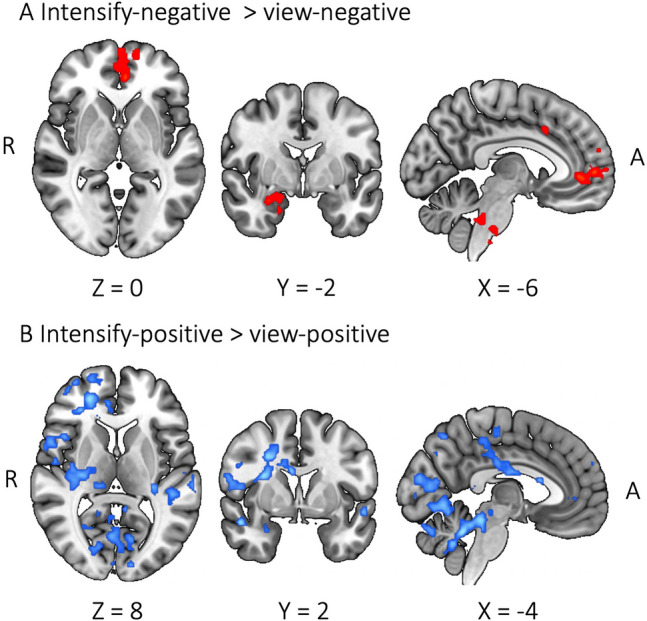
Sex differences during emotion up-regulation. Blue color displays males > females; red color displays females > males

We also observed sex differences during emotion up-regulation. Contrasting the intensify-negative condition against the view-negative condition showed increased BOLD activity in the frontal pole, paracingulate gyrus, amygdala, and cerebellum (crus I) of females compared to males (Fig. 5A), while no significant clusters were found for males versus females. The contrast of intensify-positive against view-positive revealed increased BOLD activity in broad brain regions including the frontal pole, ACC, PCC, paracingulate gyrus, precentral gyrus, precuneus cortex, and cerebellum (I–IV) of males compared to females (Fig. 5B), while no significant clusters were found for females versus males.

**Fig. 6:**
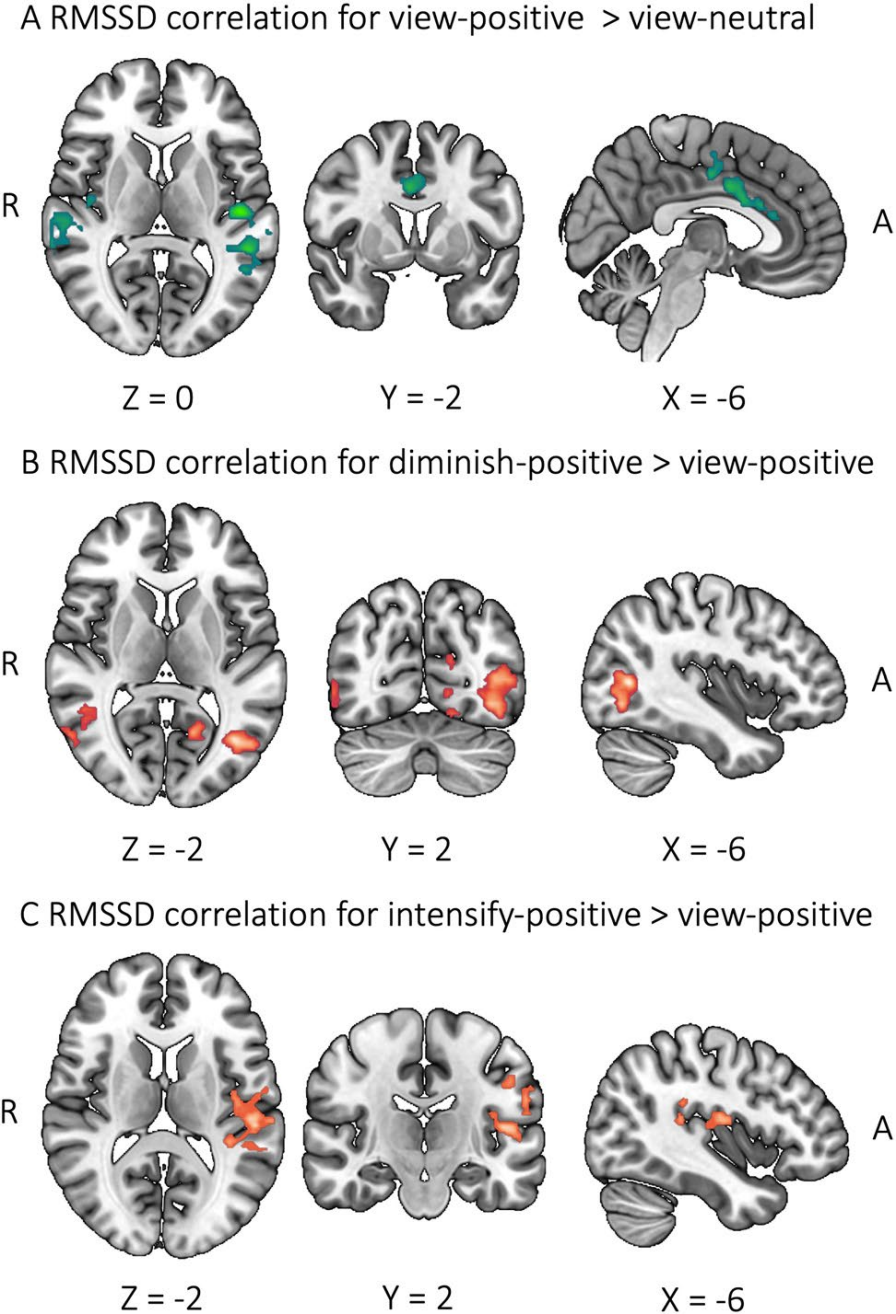
Sex differences in brain regions associated with resting RMSSD and emotion regulation. Green displays brain regions correlated with RMSSD more for males than females; orange displays brain regions correlated with RMSSD more for females than males

We found sex differences in the relationship of the neural correlates of ER and resting RMSSD. During viewing positive images against viewing neutral images, BOLD activity in the ACC and insula were correlated with RMSSD greater for males than females (Fig. 6A). During diminishing positive emotion against viewing positive emotion, BOLD activity in the lateral occipital cortex and middle temporal gyrus showed greater correlation with RMSSD for females than males (Fig. 6B). During intensifying positive emotion against viewing positive images, females relative to males had greater correlation with RMSSD in the region covering the insula, central opercular cortex, planum temporale, and Heschl’s gyrus (Fig. 6C). No other contrasts involving negative emotion showed significant clusters for correlation with RMSSD.

## Discussion

The current study investigated sex differences in the neural correlates of ER and resting HRV. We first tested sex differences in self-rated emotional intensity during regulating negative and positive emotions. We also attempted to resolve the inconsistent findings in the neuroimaging studies by having a more complete representation of valence and arousal categories in our ER task. Finally, we explored sex differences in the whole-brain regions whose activity during ER correlated with resting HRV.

We found sex differences in the subjective experience of negative and positive emotions. Self-reported ratings of emotional intensity indicated a valence by sex interaction. Females reported that they felt more intensely for negative emotion than positive emotion, but this pattern was not observed in males. Interestingly, a similar valence by sex interaction pattern appeared in the amygdala’s BOLD activity. Females showed greater amygdala activity for negative emotion than positive emotion, while males did not show such a pattern. This is in line with prior findings suggesting that females are more sensitive to negative stimuli than males (Deng et al. 2016; Gohier et al. 2013).

While amygdala activity showed no overall sex difference across different regulation conditions, we observed sex differences in PFC activity. Females compared to males showed greater PFC activity across all three regulation conditions (Fig. 2D–F). Considering no corresponding sex difference in amygdala activity, the greater PFC activity in females than males appear to support the efficiency framework where males put less effort (i.e., less PFC activity) for the similar outcome (i.e., similar amygdala activity) (McRae et al. 2008; Whittle et al. 2011). However, our results suggest that valence might drive the sex differences in amygdala activity during ER. For negative emotion, males better down-regulated the amygdala’s activity compared to viewing while females better up-regulated its activity (Fig. 2A–C). For positive emotion, males better up-regulated amygdala activity compared to viewing (Fig. 2B–C). Given the overall high PFC activity in females, the efficiency framework for males might work when down-regulating negative emotion and up-regulating positive emotion, but males do not appear to effectively down-regulate positive emotion and up-regulate negative emotion at least as reflected in terms of changes in their amygdala activity.

With the whole-brain analysis, we examined whether males and females rely on different brain regions during ER. While passively viewing negative images compared to neutral images, males relative to females showed significant BOLD signal increase in the mPFC regions (Fig. 3A). Similar mPFC areas were activated for males compared to females during down-regulating versus viewing positive emotion. The region was also activated for females compared to males during up-regulating versus viewing negative emotion. With males’ mPFC activation during viewing, we could infer that males might be automatically engaged with regulating negative emotion (Mauss et al. 2007; McRae et al. 2008). However, the finding that neither up- nor down-regulation of negative emotion activated the mPFC region for males compared to females can argue against this interpretation. An alternative explanation is that the mPFC area during viewing negative images might simply be involved in maintaining or appraising the emotion rather than implicitly regulating emotion (Etkin et al. 2011; Waugh et al. 2014).

Males and females did not show differences in resting RMSSD. However, we observed greater high-frequency power for females versus males (Table 3). The results are aligned with the findings of a meta-analysis (Koenig and Thayer 2016), indicating a relative dominance of parasympathetic activity in females. The neural correlates of resting RMSSD in relation to ER showed sex differences only when experiencing or regulating positive emotion (Fig. 6). Positive mood is generally associated with greater resting HRV (Schwerdtfeger and Gerteis 2014), and positive emotion increases HRV in the moment (Wu et al. 2019). While viewing positive images compared to neutral images, the posterior insula and ACC showed a stronger correlation with RMSSD for males than females (Fig. 6A). But RMSSD correlation in the posterior insula and central opercular cortex was stronger for females than males during intensifying positive emotion compared to viewing (Fig. 6C). In addition, the posterior insula was not shown during diminishing positive emotion compared to viewing for either sex (Fig. 4B). Brain regions including or near the posterior insula and opercular cortex have been reported to be associated with regulation of positive emotion (Li et al. 2018; Mak et al. 2009). The posterior insula also plays a role in autonomic control during affective tasks (Beissner et al. 2013), exerting influence on heart rate changes (Oppenheimer and Cechetto 2016). While viewing positive images without a conscious regulation effort, males might have a stronger coupling than females between insular BOLD activity and resting HRV, which suggests a tighter control of the autonomic system when passively experiencing positive emotion (Fig. 6A). This coupling became tighter for females than males when intensifying positive emotion (Fig. 6C).

The current study is limited because it did not control for menstrual phases in females, which might have affected reactivity to emotional stimuli (Sakaki and Mather 2012) and emotion regulation (Dan et al. 2019). Prior studies suggest that phase-dependent ovarian hormone levels can lead to different activity patterns in the amygdala and PFC regions (Toffoletto et al. 2014). During the luteal compared to follicular phase, amygdala reactivity to emotional faces was heightened (Gingnell et al. 2012) and dorsolateral PFC activity was increased with regulating response to positive words (Amin et al. 2006). Future research should incorporate hormonal phases in females to explain sex differences in amygdala and PFC activity levels (e.g., Goldstein et al. 2005, 2010; Jacobs et al. 2014; Nasseri et al. 2020).

In conclusion, our study revealed sex differences in brain activity during regulating emotion. The valence by sex interaction in self-reported emotional intensity and amygdala activity indicated that valence might be an important factor in sex differences in ER. While less PFC activity in males during ER appeared to support the idea that males are more efficient in down-regulating negative emotion, overall amygdala activity patterns for positive emotion across the three regulation conditions suggest that females might be more effective in modulating positive emotion. This calls for the reevaluation of the framework positing that ER is more efficient in males by taking into account valence. The whole-brain analysis also suggests that HRV at rest is related to the role of the posterior insula when experiencing positive emotion.

## Data Availability

Data supporting the findings of this study is made publicly available at OpenNeuro.
