# Correction to: Sex Differences in Neural Correlates of Emotion Regulation in Relation to Resting Heart Rate Variability

**DOI:** 10.1007/s10548-024-01069-9

**Published:** 2024-08-06

**Authors:** Jungwon Min, Julian Koenig, Kaoru Nashiro, Hyun Joo Yoo, Christine Cho, Julian F. Thayer, Mara Mather

**Affiliations:** 1 Davis School of Gerontology, University of Southern California, Los Angeles, USA; 2 Department of Psychology, University of Southern California, Los Angeles, USA; 3 Faculty of Medicine and University Hospital Cologne, Department of Child and Adolescent Psychiatry, Psychosomatics and Psychotherapy, University of Cologne, Cologne, Germany; 4 Department of Psychological Science, University of California Irvine, Irvine, USA

**Correction to: Brain Topography**

10.1007/s10548-023-00974-9

In this article, the sentence beginning with “There was a significant main effect” in **“Results”** section under the heading **“Self-Rated Emotional Intensity and HRV”** has “a”, but it should be “no”.

**Incorrect Sentence**: There was a significant main effect of sex, *F*(1, 98) = 0.78, *p* = 0.38, *Δη*^2^ = 0.01 (M = 2.49 and SE = 0.06 for males; M = 2.42 and SE = 0.06 for females).

**Correct Sentence**: There was no significant main effect of sex, *F*(1, 98) = 0.78, *p* = 0.38, *Δη*^2^ = 0.01 (M = 2.49 and SE = 0.06 for males; M = 2.42 and SE = 0.06 for females).

The original article has been corrected.

